# Efficacy assessment of liposome crosslinked hyaluronic acid and standard hyaluronic acid eye drops for dry eye disease management: a comparative study employing the ocular surface analyzer and subjective questionnaires

**DOI:** 10.3389/fmed.2024.1264695

**Published:** 2024-06-26

**Authors:** José-María Sánchez-González, Concepción De-Hita-Cantalejo, María Luisa González-Rodríguez, Ana Fernández-Trueba-Fagúndez, Antonio Ballesteros-Sánchez, Clara Martinez-Perez, Romina Caro-Díaz, Carla Montiel Guzman, María Fernanda González-Oyarce, María Carmen Sánchez-González

**Affiliations:** ^1^Department of Physics of Condensed Matter, Optics Area, Vision Sciences Research Group (CIVIUS), Pharmacy School, University of Seville, Seville, Spain; ^2^Faculty of Pharmacy, Department of Pharmaceutical Technology, University of Seville, Seville, Spain; ^3^Department of Ophthalmology, Clínica Novovisión, Murcia, Spain; ^4^Department of Optometry, ISEC LISBOA-Instituto Superior de Educação e Ciências, Lisbon, Portugal; ^5^Department of Medical Technology with Ophthalmology and Optometry Mention, Medicine and Science School, Universidad San Sebastián, Valdivia, Chile

**Keywords:** crosslinked hyaluronic acid, hyaluronic acid, dry eye disease, liposome, lipid layer, tear film stability, ocular surface

## Abstract

**Introduction:**

Dry eye disease (DED) is a prevalent condition causing ocular discomfort and visual disturbances, often managed with artificial tears. This study aimed to assess and compare the efficacy of eye drops containing Crosslinked Hyaluronic Acid (CHA) with liposomes and crocin and standard Hyaluronic Acid (HA) for DED management.

**Methods:**

A single-blind, longitudinal study was conducted on 24 participants (48 eyes), randomized to receive one of the two treatments. Ocular health measures, including the ocular surface disease index (OSDI) and the standard patient evaluation of eye dryness (SPEED) scores, were assessed at baseline and 6 weeks post-treatment using the Ocular Surface Analyzer.

**Results:**

CHA achieved a lipid layer thickness increase of 1.29 ± 1.08 Guillon pattern degree (*p* < 0.01), FNIBUT increase 0.64 ± 0.77 s (p < 0.01), MNIBUT increase1.28 ± 4.74 s (*p* = 0.19), OSDI decrease 11.72 ± 6.73 score points (*p* < 0.01) and SPEED decrease 1.16 ± 5.05 score points (*p* = 0.27). Significant reductions in the OSDI and SPEED scores post-treatment were observed with both treatments, indicating their effectiveness.

**Conclusion:**

CHA with liposomes exhibits superior efficacy compared to standard HA eye drops in the management of DED. These findings highlight the potential for personalized treatment strategies incorporating CHA, indicating a more effective approach to DED management. However, further research is required to validate these results and investigate the long-term effects, which may pave the way for a data-driven and optimized approach to managing DED.

## 1 Introduction

Dry eye disease (DED) is a multifactorial condition characterized by insufficient tear production, excessive tear evaporation, or a combination of both, resulting in ocular discomfort and visual disturbances (1, 2). It affects a significant portion of the population, with varying degrees of severity and impact on quality of life (3). Comprehensive management of DED involves considering both the symptoms experienced by patients and the underlying etiological factors (4). The tear film, composed of a lipid layer, aqueous layer, and mucin layer, plays a crucial role in maintaining the ocular surface health and visual clarity (5). Disruptions in any of these layers can lead to tear film instability and subsequent dry eye symptoms. Addressing tear film instability is a key aspect of DED treatment, and artificial tears are commonly used to supplement the deficient tear film and alleviate symptoms (6).

Artificial tears typically consist of an aqueous base with additional molecules incorporated to enhance lubrication, viscosity, osmolarity, tolerance, and residence time on the ocular surface (7). Viscosifying agents, such as hyaluronic acid (HA), are frequently included in tear preparations to provide lubrication and prolong the contact time with the ocular surface (8). HA is a high-molecular-weight linear polymer of natural origin with excellent hydrophilic properties (9). It exhibits viscoelasticity, making it an ideal candidate for enhancing tear film stability and providing ocular surface hydration and protection (10). Moreover, HA possesses regenerative, antioxidant, and anti-inflammatory properties that can further contribute to the management of DED (7).

While standard HA eye drops have been widely used, their effectiveness may diminish over time due to rapid clearance from the ocular surface (11). To address this limitation, crosslinked HA formulations have been developed (12). Crosslinking increases the molecular density of HA, leading to prolonged retention on the ocular surface and improved longevity of its effects (13). The incorporation of crosslinked HA in tear preparations has shown promise in enhancing tear film stability and alleviating dry eye symptoms (14). Oxidative stress is recognized as one of the contributing factors in DED. Antioxidant agents have gained attention as potential therapeutic options to mitigate the effects of oxidative stress and improve treatment outcomes (15). Crocin, a natural chemical compound found in saffron flower, has been noted for its antioxidant and anti-inflammatory properties (16). In addition, crocin can increase the viscosity and mucoadhesive properties of the tear preparation on the ocular surface, further enhancing its efficacy (17).

Furthermore, tear film lipid layer (TFLL) instability is frequently observed in DED, necessitating the inclusion of lipids in tear formulations (18). Liposomes, lipid vesicles, have emerged as a suitable delivery system for replenishing the TFLL and reducing surface evaporation (19). Liposomal formulations offer advantages in terms of improving bioavailability and enhancing the transport of active ingredients, both hydrophilic and lipophilic, to the ocular tissues (20). This comparative study seeks to contribute to our understanding of the potential benefits of novel formulations incorporating liposomes, crosslinked HA, and crocin in DED management (21). The findings may shed light on improved treatment approaches that address tear film instability and TFLL instability, ultimately enhancing patient outcomes and quality of life (22).

The purpose of our research is to assess the efficacy and longevity of 0.15% crosslinked HA with liposomes and crocin to the effects of 0.15% standard HA alone. The evaluation will be conducted utilizing the Ocular Surface Analyzer to measure tear film parameters objectively and subjective questionnaires to assess patient-reported outcomes.

## 2 Materials and methods

### 2.1 Design

This prospective, longitudinal, single-blind, single-center study was conducted at the Physics of Condensed Matter, Optics area Department of the Pharmacy School, University of Seville, Spain. The study was designed in accordance with the Helsinki Declaration and received approval from the Ethics Committee Board of Andalusia.

### 2.2 Subjects

A total of 24 subjects participated in the study after providing informed consent. Eligible participants were between 18 to 30 years old and had an ocular surface disease index (OSDI) score above 5 points, indicating symptoms of dry eye disease (DED). The inclusion criteria required that subjects were healthy and had not received any previous eye treatment. Exclusion criteria included a history of eye surgery, systemic diseases, ongoing pharmacological treatment, or contact lens wear.

### 2.3 Materials

The noninvasive tear film analysis was performed using the Integrated Clinical Platform (ICP) Ocular Surface Analyzer (OSA) from SBM System® (Orbassano, Torino, Italy). The OSA combines various diagnostic tests for dry eye disease and provides a comprehensive assessment of the ocular surface (23, 24). The instrument includes features such as high-resolution imaging, multishot and movie acquisition modes, Placido disc, and NIBUT grids. Two subjective dry eye disease questionnaires, the Ocular Surface Disease Index (OSDI) and the Standard Patient Evaluation of Eye Dryness (SPEED) test, were employed.

Two eye drop formulations were studied. Eye drop A (LCHA group) contained 0.15% crosslinked hyaluronic acid sodium salt, liposomes, crocin, ethylenediaminetetraacetic (EDTA) acid sodium salt, and a 7.2 pH isotonic buffered solution. It was provided in a multidose 10 milliliter bottle (Aquoral Lipo®, distributed by ESTEVE Pharmaceuticals®, Barcelona, Spain, manufactured by Omisan Farmaceuti®, Guidonia Montecelio, Italy). Eye drop B (HA group) served as the control and consisted of 0.15% hyaluronic acid sodium salt, sodium chloride, trometamol, hydrochloric acid, and a 7.2 pH isotonic buffered solution. It was also packaged in a multidose 10 milliliter bottle (Hyabak®, Laboratories Thea, Clermont Ferrand, France).

### 2.4 Examination procedure

The study commenced with a selection phase in which subjects were included or excluded based on the predefined criteria. Randomization was performed using computer-generated random numbers, assigning participants to either eyedrop A or B. All participants were instructed to abstain from using any eye lubricants or drops for 1 week before the study. The eye drops (one drop per eye) were self-administered by the patients in their homes at two specific times: initially in the morning upon waking (around 10:00 am) and subsequently at the end of the day before sleep (around 10:00 pm). The posology was designed to maintain a 12-h interval between applications, thereby ensuring consistent treatment twice a day. Subsequently, subjective questionnaires and noninvasive examinations using the OSA were conducted to assess various tear film parameters, including limbal and bulbar redness classification, lipid layer thickness, tear meniscus height, and noninvasive break-up time (23, 24). Following the initial assessment, the subjects were re-evaluated after 6 weeks to examine the sustained effects of both eyedrops. The temperature and humidity in the examination room were carefully controlled throughout the measurements.

### 2.5 Statistical analysis

Statistical analysis was performed using SPSS statistical software (version 26.0, IBM Corp., Armonk, New York, United States). Descriptive analysis was conducted and mean ± standard deviation (SD) values were calculated. The Shapiro–Wilk test was employed to assess the normality distribution of the data. The chi-square test was used for assessing differences in qualitative variables. Within-group differences for OSA measurements were analyzed using the Wilcoxon test, while differences between eyedrop groups were assessed using the Mann–Whitney U test. Correlation analysis was performed using the Spearman Rho test. The significance level was set at 95% (*p* value <0.05). The sample size calculation was conducted using the GRANMO® calculator, with a recommended sample size of 24 subjects based on anticipated effect sizes, standard deviations, and a predetermined alpha and beta level.

## 3 Results

This analysis encompassed a dataset of 24 participants, yielding 48 eyes that were administered one of two types of artificial tear film treatments: Eyedrop A (Crosslinked Hyaluronic Acid) and Eyedrop B (Hyaluronic Acid). These treatments were evenly divided among the participants. The sample was comprised of 25% male and 75% female participants. The participants’ age ranged from 18 to 24 years, with a mean of 21.3 ± 1.6 years (Table 1).

**Table 1:** Demographics and baseline subjects’ characteristics.

Variable	0.15% Liposome-CHA (*n* = 48)	0.15% HA (*n* = 48)	*p* value
Male (%) Female (%)	6 (25.0) 18 (75.0)	12 (50.0) 12 (50.0)	0.08
Age (years)	20.67 ± 2.18 (18.00 to 24.00)	21.42 ± 0.77 (20.00 to 22.00)	0.14
Sphere refraction (D)	−0.95 ± 1.66 (−3.50 to +0.50)	0.14 ± 1.41 (−2.00 to 3.25)	0.29
Cylinder refraction (D)	−0.37 ± 0.38 (−1.25 to 0.00)	−0.45 ± 0.54 (−1.75 to 0.75)	0.35
Axis refraction (Degrees)	76.17 ± 81.16 (0.00 to 180.00)	94.00 ± 58.92 (5.00 to 175.00)	0.20
CDVA (Log MAR)	−0.04 ± 0.07 (−0.10 to +0.10)	−0.03 ± 0.08 (−0.10 to +0.10)	0.90
Schirmer (mm)	9.83 ± 8.89 (0.00 to 30.00)	15.46 ± 9.15 (0.00 to 35.00)	0.11
BUT (seconds)	6.92 ± 2.71 (3.00 to 10.00)	8.21 ± 3.90 (3.00 to 13.00)	0.24

CHA, crosslinked hyaluronic acid; HA, hyaluronic acid, NIBUT, non-invasive break-up time, OSDI, ocular surface disease index; SPEED, standard patient evaluation eye dryness.

**Figure 1:**
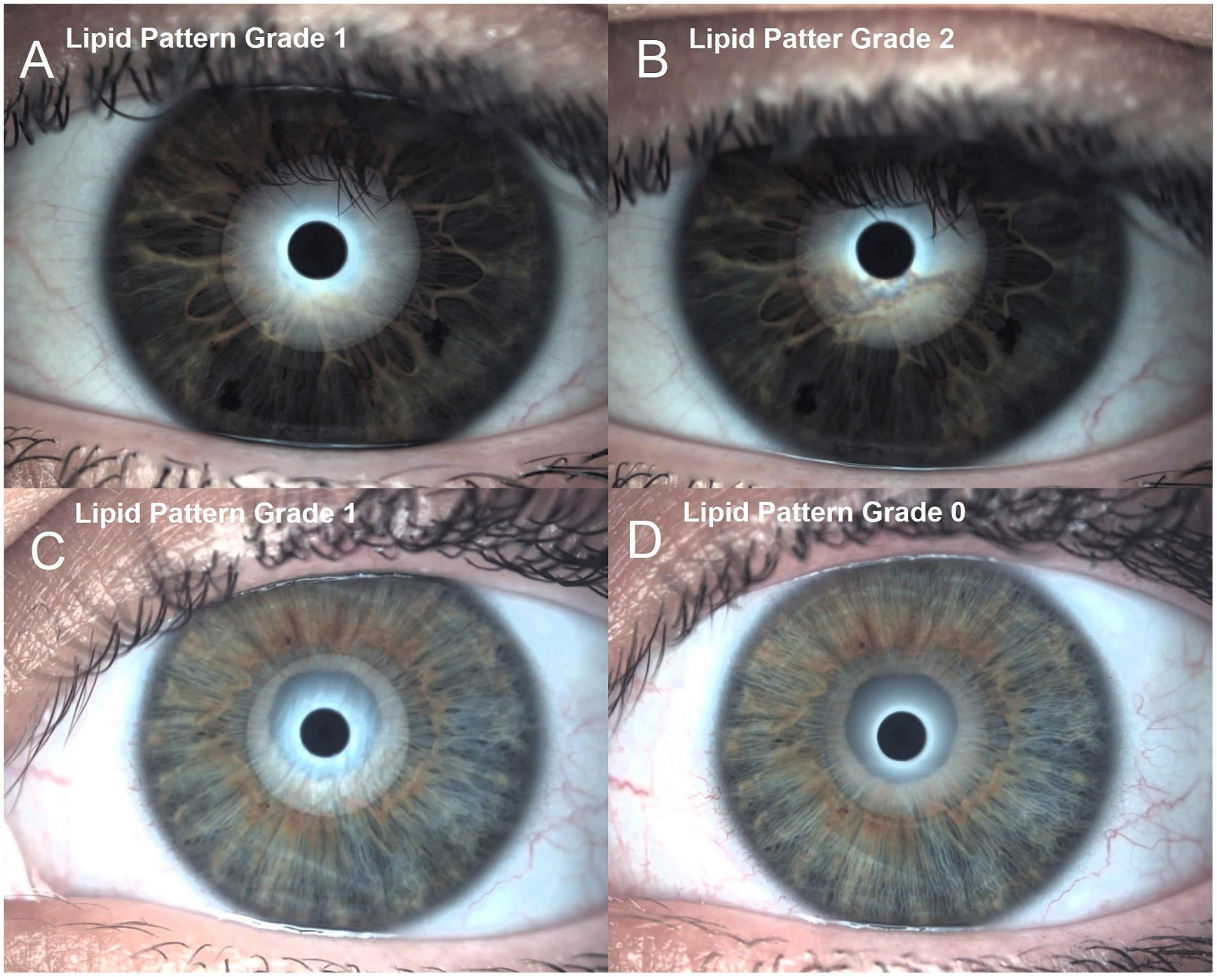
Interferometry lipid pattern differences between previous eye drop instillation and after instillation. **(A)** 0.15% liposome crosslinked hyaluronic acid group previous to eye drop instillation. **(B)** 0.15% liposome crosslinked hyaluronic acid group after treatment. **(C)** Standard 0.15% hyaluronic acid group previous to eye drop instillation. **(D)** Standard 0.15% hyaluronic acid group posterior to eye drop treatment. This figure is a representation of the change in lipid layer extract from Sánchez-González et al. (12).

The pre-treatment and post-treatment ocular surface and subjective questionnaire variables differences between CHA and HA group were presented in Table 2. In a longitudinal analysis (Table 3), CHA group achieve the following results: conjunctival redness classification increase 0.62 ± 0.96 Efron degrees (*p* < 0.01), lipid layer thickness increase 1.29 ± 1.08 Guillon pattern degree (p < 0.01), tear meniscus height not changed with 0.00 ± 0.04 millimeters variation (*p* = 0.53), FNIBUT increase 0.64 ± 0.77 s (*p* < 0.01), MNIBUT increase 1.28 ± 4.74 s (*p* = 0.19), OSDI decrease 11.72 ± 6.73 score points (*p* < 0.01) and SPEED decrease 1.16 ± 5.05 score points (*p* = 0.27). Regarding HA group the results were conjunctival redness classification increase 0.58 ± 0.88 Efron degrees (*p* < 0.01), lipid layer thickness decrease 1.00 ± 0.97 Guillon pattern degree (p < 0.01), tear meniscus height slightly decrease 0.03 ± 0.02 millimeters (*p* < 0.01), FNIBUT decrease 0.35 ± 1.66 s (*p* = 0.30), MNIBUT decrease 0.15 ± 6.76 s (*p* = 0.91), OSDI decrease 26.77 ± 28.41 score points (p < 0.01) and SPEED decrease 3.33 ± 6.61 score points (*p* = 0.02). Interferometry lipid pattern differences between previous eye drop instillation and after instillation is presented in Figure 1. In Figures 1A,B, the lipid pattern change with the LCHA group is presented. It can be observed that the lipid pattern increases from grade 1 to grade 2, indicating an improvement. In contrast, Figures 1C,D shows the lipid pattern change with the HA alone group, where the lipid pattern decreases from grade 1 to grade 0, indicating a reduction in the lipid layer.

**Table 2:** Ocular surface analyzer and subjective questionnaire comparison previous and after both eyedrop instillation.

Variable		0.15% Liposome-CHA (*n* = 48)	0.15% HA (*n* = 48)	*p* value
Baseline	Conjunctival redness (Efron scale)	0.67 ± 0.76 (0.00 to 2.00)	0.83 ± 0.56 (0.00 to 2.00)	0.39
	Lipid layer thickness (Guillon pattern)	1.00 ± 0.83 (0.00 to 2.00)	1.62 ± 0.71 (1.00 to 3.00)	< 0.01
	Tear meniscus height (Millimeters)	0.19 ± 0.02 (0.14 to 0.23)	0.20 ± 0.15 (0.18 to 0.23)	0.01
	First NIBUT (seconds)	4.45 ± 0.40 (3.95 to 5.10)	5.30 ± 1.42 (3.72 to 8.04)	0.09
	Mean NIBUT (seconds)	10.85 ± 3.62 (6.25 to 19.50)	11.33 ± 3.77 (8.76 to 18.89)	0.66
	OSDI (Score points)	21.33 ± 12.23 (6.81 to 41.66)	40.87 ± 27.13 (7.50 to 77.27)	< 0.01
	SPEED (Score points)	7.50 ± 5.09 (2.00 to 17.00)	10.41 ± 6.14 (2.00 to 19.00)	0.08
After six-weeks	Conjunctival redness (Efron Scale)	1.29 ± 0.46 (1.00 to 2.00)	1.41 ± 0.65 (0.00 to 2.00)	0.44
	Lipid layer thickness (Guillon Pattern)	2.29 ± 0.95 (0.00 to 4.00)	0.62 ± 0.57 (0.00 to 2.00)	< 0.01
	Tear meniscus height (Millimeters)	0.18 ± 0.03 (0.11 to 0.23)	0.16 ± 0.02 (0.07 to 0.22)	0.04
	First NIBUT (seconds)	5.09 ± 0.59 (3.88 to 5.84)	4.94 ± 0.59 (3.08 to 8.72)	0.39
	Mean NIBUT (seconds)	12.13 ± 3.37 (4.34 to 17.32)	11.18 ± 4.73 (5.40 to 26.38)	0.42
	OSDI (Score points)	8.04 ± 6.43 (0.00 to 18.00)	12.75 ± 3.56 (8.00 to 20.00)	0.01
	SPEED (Score points)	6.33 ± 2.77 (0.00 to 10.00)	7.08 ± 1.79 (4.00 to 10.00)	0.27

CHA, crosslinked hyaluronic acid; HA, hyaluronic acid, NIBUT, non-invasive break-up time, OSDI, ocular surface disease index; SPEED, standard patient evaluation eye dryness.

**Table 3:** Longitudinal analysis after six weeks of treatment.

Variable	0.15% Liposome-CHA (*n* = 48)		p value	0.15% HA (*n* = 48)		*p* value
	Baseline	After six-weeks		Baseline	After six-weeks	
Conjunctival Redness (Efron Scale)	0.67 ± 0.76 (0.00 to 2.00)	1.29 ± 0.46 (1.00 to 2.00)	< 0.01	0.83 ± 0.56 (0.00 to 2.00)	1.41 ± 0.65 (0.00 to 2.00)	< 0.01
Lipid Layer Thickness (Guillon Pattern)	1.00 ± 0.83 (0.00 to 2.00)	2.29 ± 0.95 (0.00 to 4.00)	< 0.01	1.62 ± 0.71 (1.00 to 3.00)	0.62 ± 0.57 (0.00 to 2.00)	< 0.01
Tear Meniscus Height (Millimeters)	0.19 ± 0.02 (0.14 to 0.23)	0.18 ± 0.03 (0.11 to 0.23)	0.53	0.20 ± 0.15 (0.18 to 0.23)	0.16 ± 0.02 (0.07 to 0.22)	< 0.01
First NIBUT (seconds)	4.45 ± 0.40 (3.95 to 5.10)	5.09 ± 0.59 (3.88 to 5.84)	< 0.01	5.30 ± 1.42 (3.72 to 8.04)	4.94 ± 0.59 (3.08 to 8.72)	0.30
Mean NIBUT (seconds)	10.85 ± 3.62 (6.25 to 19.50)	12.13 ± 3.37 (4.34 to 17.32)	0.19	11.33 ± 3.78 (8.76 to 18.98)	11.18 ± 4.73 (5.40 to 26.38)	0.91
OSDI (Score points)	21.33 ± 12.23 (21.33 to 12.23)	8.04 ± 6.43 (0.00 to 18.00)	< 0.01	40.87 ± 27.13 (7.50 to 77.27)	12.75 ± 3.56 (8.000 to 20.00)	< 0.01
SPEED (Score points)	7.50 ± 5.09 (2.00 to 17.00)	6.33 ± 2.77 (0.00 to 10.00)	0.27	10.41 ± 6.14 (2.00 to 19.00)	7.08 ± 1.79 (4.00 to 10.00)	0.02

CHA, crosslinked hyaluronic acid; HA, hyaluronic acid, NIBUT, non-invasive break-up time, OSDI, ocular surface disease index; SPEED, standard patient evaluation eye dryness.

A correlation analysis was conducted to understand the relationships among the variables in the dataset. The pre-treatment OSDI and SPEED scores were found to be strongly correlated (*r* = 0.64, *p* < 0.0001), indicating that participants with a higher disease index score also had a higher dryness score. This correlation was consistent even after the treatment, with the post-treatment OSDI and SPEED scores also exhibiting a strong correlation (*r* = 0.96, *p* = 0.0028).

## 4 Discussion

The primary objective of this study was to examine the effects of two distinct artificial tear film treatments, Eyedrop A (Crosslinked hyaluronic acid with liposomes and crocin) and Eyedrop B (Hyaluronic Acid), on several ocular health metrics. The results gleaned from this investigation shed light on the comparative effectiveness of these treatments, which is reflected in the significant improvements in ocular health measures post-treatment. Particularly, the ocular surface disease index (OSDI) and the standard patient evaluation of eye dryness (SPEED) scores experienced considerable reductions, underscoring the efficacy of both eyedrops in managing dry eye disease. Interestingly, the study also revealed patterns related to the type of eyedrop treatment, suggesting that the specific kind of treatment can potentially influence ocular health outcomes. As we further explore these results in the upcoming sections, we will address their potential implications for clinical practice, the limitations of the current study, and avenues for future research.

The study by Yu et al. (25) explored the use of chemically crosslinked high molecular weight hyaluronic acid as a long-acting ocular surface lubricant. They found that the soft hydrogel created from crosslinked hyaluronic acid exhibited unique rheological properties similar to natural soft hydrogels, and it showed potential in improving the clinical signs of dry eye in animal and canine clinical studies. Posarelli et al. (26) highlighted the superiority of HA in increasing the viscosity and stability of the tear film compared to other tear supplements. They emphasized the importance of modifying HA to enhance its properties and adapt it to different therapeutic aims. The trend in pharmacology is to crosslink HA to improve its bioavailability and resistance to degradation, which has shown promise in providing better ocular comfort for patients with dry eye disease (27).

Our study evaluated the effects of CHA and HA on various parameters related to dry eye disease. In the CHA group, we observed an increase in conjunctival redness classification and lipid layer thickness, indicating a potential improvement in ocular surface health. The increase in FNIBUT and decrease in OSDI score further support the positive therapeutic effects of CHA. However, tear meniscus height and MNIBUT did not show significant changes. Interestingly, the HA group exhibited different results compared to the CHA group. In the HA group, conjunctival redness classification and tear meniscus height showed slight changes, while lipid layer thickness decreased. FNIBUT and OSDI score decreased, suggesting a potential improvement in tear film stability and subjective dry eye symptoms. However, MNIBUT did not show significant changes. These findings are consistent with previous studies. Fezza et al. (28) conducted a study on the safety and efficacy of a new crosslinked hyaluronic acid gel occlusive device for dry eyes. They reported improvements in corneal fluorescein staining, Schirmer’s test, tear breakup time, and tear meniscus height after the application of the gel occlusive device. Similarly, Cagini et al. (29) compared the stability of the tear film after instillation of eye drops containing HA or crosslinked HA in patients with Sjögren syndrome-related dry eye. They found that crosslinked HA as a tear supplement provided better stability to the tear film, especially in patients with dry eyes. The study by Roskowska et al. (13) evaluated the clinical efficacy of an ophthalmic solution containing crosslinked hyaluronic acid, trehalose, liposomes, and sterylamine in subjects with moderate to severe dry eye disease. They reported significant improvements in symptoms, tear breakup time, and corneal and conjunctival staining, supporting the effectiveness of crosslinked hyaluronic acid in treating dry eye. Our study also contributes to the understanding of the mucoadhesive properties of HA. Guarise et al. (9) demonstrated that the ocular residence time of HA formulation is correlated with its mucoadhesive properties. High molecular weight HA showed better mucoadhesive performance compared to crosslinked HA and other gelling agents.

Several studies have investigated the efficacy and safety of different formulations containing CHA for the treatment of dry eye disease. Postorino et al. (11) conducted a randomized controlled study comparing a collyrium based on crosslinked HA with coenzyme Q10 (CoQ10) to hyaluronic acid alone. They found that the combination of crosslinked HA with CoQ10 showed greater effectiveness in reducing dry eye symptoms, as indicated by improved scores in the Ocular Surface Disease Index (OSDI), corneal staining, and meibomian gland assessment. Similarly, Fallacara et al. (15) investigated the re-epithelialization ability of crosslinked HA formulations and observed no cellular toxicity, restoration of epithelium integrity, and increased viability compared to the control. These findings suggest the potential of crosslinked HA as a therapeutic agent for promoting corneal epithelial wound healing. Additionally, Tredici et al. (10) evaluated an ophthalmic solution containing crosslinked HA, CoQ10, and vitamin E TPGS in professional swimmers exposed to chlorinated water. Their results demonstrated significant improvements in tear film breakup time, corneal and conjunctival staining, and Ocular Surface Disease Index scores.

Moreover, the cytoprotective effects of HA in oxidative and inflammatory processes are worth mentioning. Ali et al. (7) reported that HA combined with crocin exerted antioxidant and anti-inflammatory effects in human corneal epithelial cells. This highlights the potential of HA in mitigating the underlying pathogenic processes associated with dry eye disease. Our study supports the therapeutic benefits of crosslinked hyaluronic acid in the management of dry eye disease. The unique rheological properties and improved stability of the tear film associated with crosslinked hyaluronic acid make it a promising treatment option.

### 4.1 Limitations and future directions

Despite the insightful findings, this study is not without its limitations. Primarily, the small sample size could have increased the risk of overfitting in the predictive models, potentially limiting the generalizability of the results. One limitation is the absence of recorded data on the temperature and humidity of the examination room, which could influence the outcomes of dry eye assessments. Additionally, the study design did not account for potential confounding factors that could affect ocular health measures, such as participants’ lifestyle, environmental factors, or medical history. Therefore, while the results provide a preliminary understanding of the effects of the two eyedrop treatments, caution should be exercised in interpreting the findings.

Another limitation is the lack of statistically significant improvements in SPEED scores for the CHA treatment group. While both treatments showed a reduction in the score, only HA demonstrated statistical significance. Future research with a larger and more diverse sample size is needed to validate the present findings. Longitudinal studies could provide insights into the long-term effects of the treatments, while controlled trials could further ascertain the efficacy of each eyedrop type. It could also be beneficial to explore the potential interaction effects between variables, such as whether the effect of the treatment varies depending on the participant’s sex or initial ocular health status.

Our research primarily focused on OSDI and SPEED scores as outcome measures. Including other techniques or clinical assessment methods could provide a more robust comparative analysis between CHA and HA. Future work could incorporate a variety of assessment methods beyond OSDI and SPEED scores to offer a more comprehensive view of treatment efficacy.

A final limitation is the inclusion of data from both eyes of each subject. Future studies should consider analyzing only one eye per individual, selected either randomly or consistently, to enhance the robustness of the findings.

Future research should focus on analyzing the anti-inflammatory and antioxidant properties of CHA. While our study demonstrated CHA’s efficacy in DED management, further investigations are needed to understand its underlying mechanisms. Molecular and cellular studies can elucidate CHA’s therapeutic pathways, and long-term research on oxidative stress markers will provide deeper insights into its benefits for DED treatment.

Implement prior training for the correct administration of eye drops and establish defined instillation schedules. Additionally, implement intermediate follow-ups, such as at 3 weeks, and consider an evaluation focusing on treatment adherence, symptoms, and any adverse effects or reactions to the eye drops, if present (30).

### 4.2 Implications for clinical practice

The findings of this study have several potential implications for clinical practice. The significant improvement in ocular health measures following the treatment with both types of eyedrops suggests that these treatments could be effectively used to manage symptoms in patients with dry eye disease. The observed differences in the effects of the two eyedrops underscore the importance of considering the specific type of treatment in clinical decision-making.

Furthermore, the significant associations between various ocular health measures and participant characteristics, such as sex, highlight the need for a personalized approach to treatment. Understanding these relationships could aid clinicians in predicting the likely response to treatment and tailoring the treatment plan to the individual patient’s characteristics and needs.

Finally, the predictive models developed in this study, despite their limitations, represent a step toward a more data-driven approach to patient care. With further validation and refinement, such models could potentially be used to predict treatment outcomes and guide treatment decisions in clinical practice.

## 5 Conclusion

Our findings suggest that eye drops containing CHA, liposomes, and crocin may offer advantages in managing DED, yet the evidence varies across assessed parameters. Notably, while symptom improvements were observed for both treatments, only the HA treatment showed statistically significant changes in SPEED scores. Therefore, these promising results should be interpreted with caution. Future studies should aim for a comprehensive assessment, utilizing additional techniques and extending clinical evaluation periods to draw more definitive conclusions about the comparative efficacies of CHA and HA in DED management. This research could pave the way for a more data-driven and optimized approach to treating DED.

## Data availability statement

The raw data supporting the conclusions of this article will be made available by the authors, without undue reservation.

## Ethics statement

The studies involving humans were approved by the Ethics Committee Board of Andalusia. The studies were conducted in accordance with the local legislation and institutional requirements. The participants provided their written informed consent to participate in this study. Written informed consent was obtained from the individual(s) for the publication of any potentially identifiable images or data included in this article.

## Author contributions

J-MS-G: Conceptualization, Investigation, Methodology, Supervision, Writing – original draft, Writing – review & editing. CD-H-C: Conceptualization, Investigation, Methodology, Supervision, Writing – original draft, Writing – review & editing. MG-R: Writing – review & editing, Methodology, Supervision, Conceptualization, Investigation. AF-T-F: Conceptualization, Investigation, Methodology, Supervision, Writing – original draft, Writing – review & editing. AB-S: Conceptualization, Investigation, Methodology, Supervision, Writing – original draft, Writing – review & editing. CM-P: Writing – review & editing, Data Curation, Conceptualization, Validation, Investigation. RC-D: Writing – review & editing, Methodology, Supervision, Conceptualization, Validation. CM-G: Writing – review & editing, Methodology, Supervision, Conceptualization, Validation. MG-O: Writing – review & editing, Methodology, Supervision, Validation. MS-G: Conceptualization, Investigation, Methodology, Supervision, Writing – original draft, Writing – review & editing.
